# A Novel *Escherichia coli* O157:H7 Clone Causing a Major Hemolytic Uremic Syndrome Outbreak in China

**DOI:** 10.1371/journal.pone.0036144

**Published:** 2012-04-27

**Authors:** Yanwen Xiong, Ping Wang, Ruiting Lan, Changyun Ye, Hua Wang, Jun Ren, Huaiqi Jing, Yiting Wang, Zhemin Zhou, Xuemei Bai, Zhigang Cui, Xia Luo, Ailan Zhao, Yan Wang, Shaomin Zhang, Hui Sun, Lei Wang, Jianguo Xu

**Affiliations:** 1 State Key Laboratory for Infectious Disease Prevention and Control, National Institute for Communicable Disease Control and Prevention, Changping, Beijing, China; 2 School of Biotechnology and Biomolecular Sciences, University of New South Wales, Sydney, New South Wales, Australia; 3 Jiangsu Provincial Center for Disease Control and Prevention, Nanjing, Jiangsu Province, China; 4 Anhui Provincial Center for Disease Control and Prevention, Hefei, Anhui Province, China; 5 College of Life Sciences, Nankai University, Tianjin, China; Institute for Genome Sciences, University of Maryland School of Medicine, United States of America

## Abstract

An *Escherichia coli* O157:H7 outbreak in China in 1999 caused 177 deaths due to hemolytic uremic syndrome. Sixteen outbreak associated isolates were found to belong to a new clone, sequence type 96 (ST96), based on multilocus sequence typing of 15 housekeeping genes. Whole genome sequencing of an outbreak isolate, Xuzhou21, showed that the isolate is phylogenetically closely related to the Japan 1996 outbreak isolate Sakai, both of which share the most recent common ancestor with the US outbreak isolate EDL933. The levels of IL-6 and IL-8 of peripheral blood mononuclear cells induced by Xuzhou21 and Sakai were significantly higher than that induced by EDL933. Xuzhou21 also induced a significantly higher level of IL-8 than Sakai while both induced similar levels of IL-6. The expression level of Shiga toxin 2 in Xuzhou21 induced by mitomycin C was 68.6 times of that under non-inducing conditions, twice of that induced in Sakai (32.7 times) and 15 times higher than that induced in EDL933 (4.5 times). Our study shows that ST96 is a novel clone and provided significant new insights into the evolution of virulence of *E. coli* O157:H7.

## Introduction

Enterohemorrhagic *Escherichia coli* (EHEC) O157:H7 was first identified as etiological agent of bloody diarrhea in the early 1980s and has since been recognized worldwide as a cause of food- and waterborne infectious diseases [1]. It may also lead to the development of hemolytic uremic syndrome (HUS), an infection sequelae characterised by hemolysis and renal failure which can lead to long-term kidney damage or fatal outcome.

*E. coli* O157:H7 has caused many outbreaks in the past three decades, with a wide range of clinical illness [2]. In 1982, the first outbreak of O157:H7 involved at least 47 cases of diarrhea in Oregon and Michigan, associated with consumption of undercooked beef patties at fast food restaurants [1]. Subsequently there were two multi-state outbreaks in the US. In 1993 an outbreak from hamburgers had 501 cases of diarrhea reported, including 151 hospitalizations (31%), 45 cases of complicated HUS (9%), and three deaths [3], while in 2006, an outbreak associated with spinach had high rates of both hospitalization (50%) and HUS (16%) [4]. Outside the US, a massive outbreak associated with consumption of white radish sprouts in Sakai, Japan in 1996 had about 7,470 school children infected, 1,000 hospitalizations for severe gastrointestinal symptoms, 100 HUS cases and three deaths [5]. A less well known massive outbreak of O157:H7 occurred in Xuzhou, China, in 1999, with 195 hospitalized patients who had clinically diagnosed HUS and 177 deaths, which has only been reported in Chinese journals [6], [7].

Despite that a large number of virulence genes in O157:H7 have been identified, the factors critical for HUS development is poorly understood. The hallmark of the disease is the production of Shiga toxins in the intestine by O157:H7 leading to the damage of the endothelial cells and potential HUS. Two forms of the Shiga toxin, Stx1 and Stx2, are known with the latter being more cytotoxic which may increase the risk of developing HUS [8], [9]. Variants of *stx*2 have been observed in O157:H7 [10] and strains carry one or two *stx2* alleles are more likely to cause HUS [11], [12]. It has also been shown that the Shiga toxin genotype rather than the amount of Shiga toxin or the cytotoxicity of Shiga toxin *in vitro* correlates with the appearance of HUS [12].

A recent study suggests that the 2006 Spinach outbreak strain has evolved to higher virulence [13], which has increased in frequency in recent years in the US, and is significantly more likely to be associated with HUS [2]. In this study we investigated the genomic and phenotypic properties of the 1999 China outbreak associated O157:H7 strains. We sequenced the genome of the 1999 China outbreak strain Xuzhou21, and compared it with the genomes of the 1996 Japan outbreak strain Sakai, the 1982 and 2006 US outbreak strains EDL933 and TW14359. We show that Xuzhou21 carries a highly inducible *stx2* expression and has the ability to provoke significantly elevated inflammatory response.

## Materials and Methods

### Laboratory investigation of the outbreak

Isolation of O157:H7 from patient's fecal sample was done using Sorbitol-MacConkey agar [14]. Sorbitol non-fermenting colonies were selected for biochemical and agglutination tests with O157 and H7 antisera. *E. coli* O157:H7 was isolated from other sources by enrichment using anti-O157 coated immunomagnetic beads and plating on Sorbitol-MacConkey agar [14].

### Bacterial isolates

The O157:H7 strains used in this study were isolated from feces of human patients and animals between 1988 and 2005 from Xuzhou City, Jiangsu province (80 strains) and the neighboring Anhui (25 strains), Henan (nine strains) and Shandong (six strains) provinces. Four isolates from other parts of China (Yunnan province), two isolates from USA and eight isolates from Japan were also included, as well as three fully sequenced strains EDL933, Sakai and TW14359 (Table 1).

**Table 1:** Characteristics of 124 *E. coli* O157:H7 Chinese isolates used in this study.

Year	Province	Source	Sequence type#	Shiga toxin genes$
1988 (1)	Jiangsu (1)	Patient (1)	ST97 (1)	*stx1*,*stx2*
1992 (6)	Shandong (6)	Patient (4)	ST97 (1)	*stx1*,*stx2*
			ST96 (2)	*stx1*(2),*stx2*(2)
			ST24 (1)	*stx2*c
		Cattle (2)	ST97 (1)	*stx1*,*stx2*
			ST24 (1)	*stx2*c
1999 (93)	Jiangsu (68)	Patient (5)	ST96 (5)	*stx1*(5),*stx2*(5)
		Cattle (7)	ST23 (6)	*stx2*c(6)
			ST96 (1)	*stx1*,*stx2*
		Chicken (13)	ST23 (10)	*stx2*c(10)
			ST96 (2)	*stx1*(2),*stx2*(2)
			ST98 (1)	*stx1*,*stx2*
		Goat (22)	ST23 (21)	*stx2*c(21)
			ST96 (1)	*stx1*,*stx2*
		Pig (21)	ST23 (20)	*stx2*(4), *stx2*c(16)
			ST96 (1)	*stx1*, *stx2*
	Anhui (25)	Cattle (6)	ST23 (6)	*stx2*(1), *stx2*c(5)
		Chicken (6)	ST23 (6)	*stx2*c(6)
		Goat (10)	ST23 (10)	*stx2*(1), *stx2*c(9)
		Pig (3)	ST23 (3)	*stx2*c(3)
2000 (7)	Jiangsu (7)	Patient (7)	ST23 (7)	*stx2*c(7)
2001 (4)	Yunnan (4)	Food (4)	ST96 (4)	*stx1*(4),*stx2*(4)
2002 (10)	Henan (6)	Food (6)	ST23 (6)	*stx1*(2),*stx2*(6)
	Jiangsu (4)	Goat (2)	ST23 (2)	*stx2*c(2)
		Cattle (1)	ST23 (1)	*stx2*c
		Pig (1)	ST23 (1)	*stx2*c
2005 (3)	Henan (3)	Goat (3)	ST23 (3)	*stx2*c(3)

# Sequence type (ST) based on multilocus typing of 15 housekeeping genes. Number of isolates for each ST is shown in parentheses.

$ The numbers in bracket after a *stx* type or subtype is the number of isolates detected carrying that *stx*.

### Multilocus sequence typing

MLST was performed on 15 housekeeping genes (*arcA, aroE, aspC, clpX, cyaA, dnaG, fadD, grpE, icdA, lysP, mdh, mtlD, mutS, rpoS, uidA*). A detailed protocol of the MLST procedure, including allelic type and sequence type (ST) assignment methods, can be found at the EcMLST website (http://www.shigatox.net/mlst). MEGA 4.0 [15] was used to construct phylogenetic trees and eBURST [16] was used to cluster STs into clonal complexes which consist of STs differing by one of the fifteen genes typed.

### Cytokines assays

Peripheral blood mononuclear cells (PBMCs) were isolated from fresh blood from healthy individuals. PBMCs were diluted sequentially up to four-fold in a 96 well plate. Bacterial cells inactivated by heat at 56°C for one hour were added to the PBMCs and incubated for four hours. IL-6 and IL-8 were assayed by use of Fluorokine MAP Multiplex Human Cytokine Panel A of the Luminex 100 Analyzer with the X-Y Platform (Luminex, USA).

### *stx2* expression assays

Cultures were grown in Luria-Bertani medium at 37°C with shaking. For induction of the Stx2 phage, mitomycin C (BBI, American) was added to a final concentration of 0.5 µg/ml and incubated for three hours. Total RNA was extracted with RNeasy Mini Kit (Qiagen, Germany). Real-time PCR determination of gene expression was performed with the Rotor-Gene Q Real-Time PCR system (Qiagen, Germany) using a One Step SYBR® PrimeScript™ RT-PCR kit (TaKaRa, Japan), according to the manufacturer's instructions. *gapA* was used as an endogenous control. The relative level of gene expression was calculated using the 2^−ΔΔCT^ method [17]. Each sample was run in triplicate.

### Verotoxicity assay

One hundred microliter aliquots of MEM-resuspended bacteria at a multiplicity of infection (MOI) of 100 or supernatant were added in triplicate to Vero cells (10^4^ cells per well) in 96-well tissue culture plates, then incubated at 37°C in a 5% CO_2_ atmosphere. After 4, 8, 12, 20 hours, the supernatant was collected and the release of the cytoplasmic lactate dehydrogenase (LDH) was evaluated using the Cytotox96 kit (Promega, Madison, Wisconsin, USA) according to the manufacturer's instructions. The percentage of cytotoxicity was expressed as (experimental release−spontaneous release)/(maximum release−spontaneous release)×100. The spontaneous release was the amount of LDH activity in the supernatant of uninfected cells and the maximum release was that when cells were lysed with the lysis buffer.

### Genome sequencing and analysis

Chromosomal DNA from Xuzhou21 was sequenced using a 454/Roche FLX machine, according to the manufacturer's protocols, producing 318,830 reads with an average length of 410 bp that were assembled de novo into 159 contigs of at least 1,000 bp, using the 454/Roche Newbler assembly program. Gaps were closed by directed PCR and sequencing with BigDye terminator chemistry on an ABI 3730 capillary sequencer. To avoid sequence miscalls in homopolymeric tracts, all questionable sites and the sites different from other *E. coli* O157 strains were checked by resequencing of PCR products using an ABI 3730. Genome annotation and comparison were done as described previously [18]. The collinear blocks of the O157:H7 genomes were determined using BLASTN. Then the alignment within each of the block was obtained using Mauve [19]. The generation of the final plot, identification of recombination segments, and allocation of SNPs to specific lineages were done similar to the method used in the study of *Vibrio cholerae* by Feng *et al.* [20].

The genome sequence and pO157 sequences of Xuzhou21 have been deposited in the GenBank database under accession numbers CP001925 and CP001926 respectively.

## Results

### Epidemiological investigation of the outbreak

**Figure 1:**
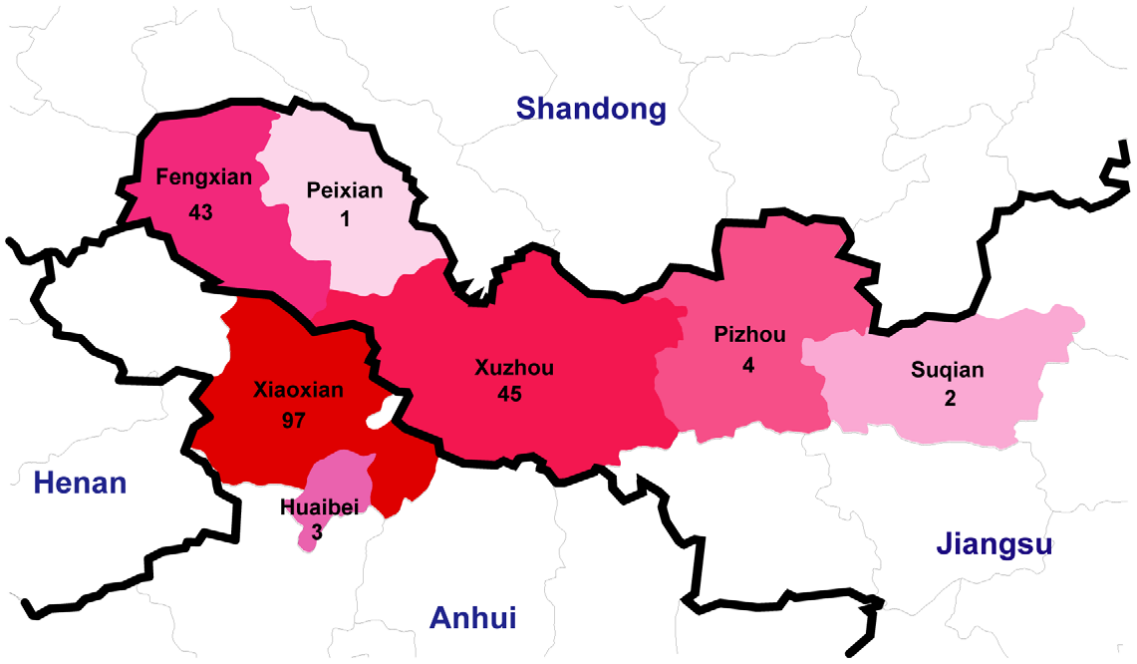
Geographic distribution of HUS cases of the 1999 outbreak in Jiangsu and neighbouring Anhui province.

The O157:H7 outbreak occurred between April and September and peaked in June, 1999 with 195 HUS cases and 177 deaths from 52 villages of seven counties in Jiangsu and neighboring Anhui province (Table 2, Figure 1 and Figure S1). Of the 195 cases, 167 (85.6%) were over 50 years old with only two less than 20 years old and 121 (62.1%) were female. The National Institute for Communicable Disease Control and Prevention, China CDC, commenced the outbreak investigation on June 28, 1999. Three and two strains of O157:H7 were isolated in Xuzhou city from fecal screening of 30 HUS and 25 diarrhea patients respectively. Thirty six sera collected from 42 HUS patients (85.7%) tested positive for IgG against EHEC-hemolysin or O157 lipopolysaccharide. Thus both bacterial and serological data confirmed that the outbreak was caused by O157:H7. The source of the infection was investigated using a case-control sample of 146 HUS patients and 840 healthy individuals, matched in age, sex and residence. No hand-washing before eating, consumption of fruits or vegetables without washing, consumption of leftover foods without heating, no fly-net cover for foods and high density of flies in kitchen were found to be statistically associated with infected patients. Using magnetic beads coated with antibodies against the O antigen, O157:H7 was isolated from six of 67 (9.0%) fly specimens, four of 74 (5.4%) raw meats and three of 83 (3.6%) cooked meats. O157:H7 was also isolated from live animals including 32 of 189 (16.9%) cattle, 50 of 605 (8.3%) pigs, 91 of 590 (15.4%) goats and 52 of 604 (8.6%) chickens raised in courtyards of families with and without HUS patients in the same villages.

**Table 2:** The age distribution of HUS patients of 1999 China O157:H7 outbreak.

	Jiangsu Province		Anhui Province		Total	
Age group	No. of cases	No. of fatal cases	No. of cases	No. of fatal	No. of cases	No. of fatal
<30	2	1	2	2	4	3
30–39	6	5	1	1	7	6
40–49	3	3	4	4	7	7
50–59	11	8	10	9	21	17
60–69	20	18	25	25	45	43
70–79	39	34	40	36	79	70
≥80	14	14	18	17	32	31
Total	95	83	100	94	195	177

### The 1999 China outbreak isolates belonged to a novel clone ST96

**Figure 2:**
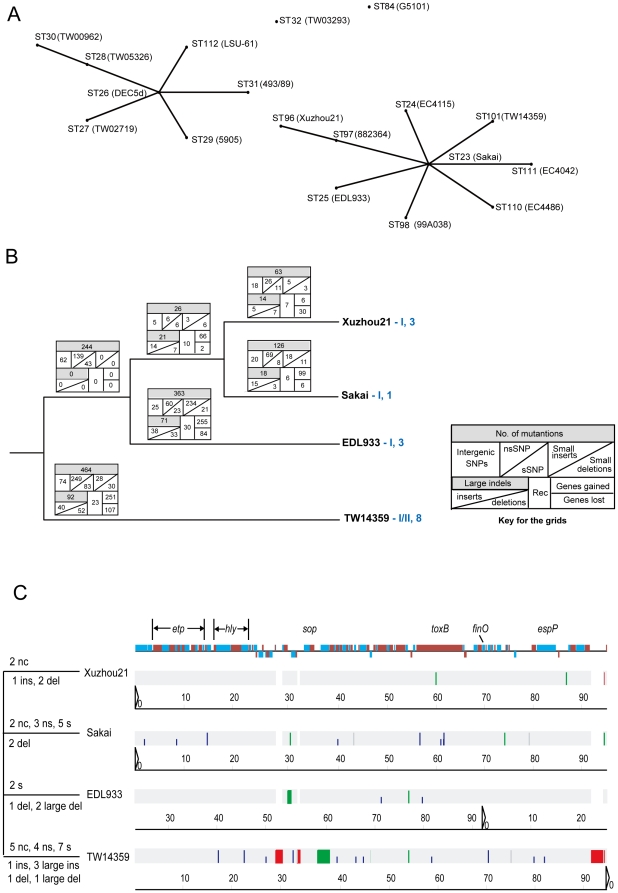
Genetic relationships of O157:H7 isolates. (A): eBURST analysis of the O157:H7 sequence types. Majority of the STs belong to two clonal complexes which was defined based on one out 15 genes being different. Names after ST in brackets are strain names. The recently sequenced strains [22] and their ST assignments are as follows: TW14588: ST23; EC4009, EC4127, EC4191, EC4401, EC4045, EC4076, EC869, 1125, FRIK2000, FRIK966: ST24; EC536: ST97; EC4486: ST110; EC4042: ST111; and LSU-61: ST112. (B). Genomic relationships of Xuzhou21 with other O157:H7 strains. The evolutionary relationship of the four genome sequenced strains of O157:H7 were determined using mutational SNPs. Allocation of SNPs to specific lineages were done using *E. coli* O55:H7 CB9615 as an outgroup [48]. Changes were shown in the boxes along the branches. Rec: recombination; sSNP: synonymous SNPs; nsSNP: non-synonymous SNPs. The lineages were defined based on the LSPA6 lineages [25], [26] and clades were based on Manning *et al.* SNP typing [2]. (C): Genetic changes and relationships of the pO157 plasmids from the four strains. The bar at the top indicates coding genes in alternative colour with those transcribed in reverse direction pointing downwards. The key genes are marked above the bar. Changes in each plasmid are marked on branches on the left. Ins, del, nc, s, ns and large del denote single base insertion, single base deletion, non- coding SNP, sSNP, nsSNP, and large deletion respectively. The colours for the vertical lines on the grey strips are red for insertion, green for deletion and blue for other single base changes with shorter line for synonymous changes. The scale is in kb.

The 15-locus multilocus sequence typing (MLST) scheme developed by Whittam and colleagues (http://www.shigatox.net/ecmlst/) which offers higher resolution than the standard 7-locus MLST [21] was used to type a selection of 124 O157:H7 isolates including the five isolates from human patients from the 1999 Xuzhou outbreak and 68 isolates from other sources in the outbreak region during the outbreak period (Table 1). MLST differentiated the 124 isolates into five sequence types (STs) (ST23, ST24, ST96, ST97 and ST98). ST23 was predominant with 83.1% of the isolates. All five Xuzhou outbreak human isolates and five animal isolates from the outbreak region were found to belong to a novel ST, ST96. Two other novel STs were ST97 and ST98. The new STs have been submitted to EcMLST database [21]. The recently fully or partially sequenced O157:H7 strains [22], [23] fall into existing STs except that three strains have new ST profiles (ST110, ST111and ST112). These STs, together with nine O157 STs (ST23, ST24, ST25, ST31, ST84, ST101, ST110, ST111,ST112) from the EcMLST database [21], were analyzed by eBURST [16]. Using the definition of one gene difference out of the 15 genes as a clonal complex, eBURST analysis found that ST96 is closer to ST97 and forms a clonal complex with seven other STs including the four genome sequenced strains, EDL933, Sakai, TW14359 and EC4115 (Figure 2A).

### Genome sequencing of a 1999 China outbreak isolate Xuzhou21 reveals a clonal relationship with Japan outbreak strain Sakai

Xuzhou21 isolated from an HUS patient from the 1999 Xuzhou outbreak was selected for genome sequencing. As reported previously [24], Xuzhou21 shared the same PFGE pattern with all four other human isolates from the same outbreak and seven other animal isolates isolated during the outbreak. The Xuzhou21 genome consists of one chromosome of 5,386,223 bp and two large plasmids (pO157 and pO157_Sal). We compared Xuzhou21 with EDL933, Sakai and TW14359 genomes. The overall relationship based on full genome sequences together with changes along the branches is shown in Figure 2B. Using the method of Feng *et al.* [23], we differentiated the base changes into mutational and recombinational changes and were also able to allocate mutational differences to specific lineages using O55:H7 strain CB9615 as an outgroup. The majority of the base changes are due to mutations. There are seven recombinational events with six being less than 200 bp while one involving 7,747 bp (Table S1). The mutational SNPs separated Xuzhou21 from EDL933 and grouped it with Sakai with 17 SNPs supporting this branching order (Table S2). However two SNPs support Xuzhou21 and TW14359 as a group. Fifty five unique SNPs are carried by the Xuzhou21 genome that separate the Chinese outbreak strain from Sakai, besides the 17 branch supporting SNPs (Table S3). The genome data also allowed us to assign Xuzhou21 to lineage I of the LSPA lineage typing scheme [25], [26] and clade 3 of Manning *et al.* SNP based typing scheme [2]. It is interesting to note that Xuzhou21 is in the same clade as EDL933 although it is closer to Sakai based on genome data as none of the 17 Xuzhou21-Sakai branch supporting SNPs was in the set of Manning *et al.* SNPs.

A complete genome (EC4115) and 25 incomplete genomes of O157:H7 were recently published by Eppinger *et al.* [22]. EC4115 has been shown to be closely related to TW14359. Although EC4115 was isolated during the 2006 spinach outbreak, it may represent a strain from a separate outbreak (see Eppinger *et al.* 2011). Our comparison of EC4115 and TW14359 revealed only 46 SNPs and thus EC4115 was not used for a detailed comparison with Xuzhou21. Eppinger *et al.* [22] also identified two strains (EC4501 and TW14588) that are closely related to Sakai. We extracted the SNPs from the genome sequences of these two strains and used the SNPs common with the mutational SNPs we identified above to construct a phylogenetic tree (Figure S2). EC4501 and TW14588 remain close to Sakai and Xuzhou21 diverged earlier than the other two strains. Both EC4501 and TW14588 are in Manning *et al.* clade 2 and the genomic relationship is consistent with the clade relationship defined by Manning *et al.* [2]. Note that EC4501 seems to be far more incomplete than TW14588 with over 270 of the 902 SNPs common to the four complete genomes are missing in EC4501, which may affect the branching order of EC4501 and TW14588.

In comparison to Sakai, there are five large insertions and seven large deletions of >100 bp in Xuzhou21 with the majority of these indels located in prophage genomes. Six genes are specific to Xuzhou21 (Table 3), among which, four genes are located in the Stx2 phage genome including *ninE* and two genes encoding putative phage replication functions while the remaining two, both encoding a transposase, located together and are flanked by an integrase gene on both sides. Surprisingly 30 genes were lost in Xuzhou21 (Table 3). Most of the genes lost are phage related. Two EDL933 phages, CP-933R and CP-933T (equivalent to Sp10 and Sp13 in Sakai), are absent in Xuzhou21 (Figure S3).

**Table 3:** Genes gained and lost in Xuzhou21.

Strain(s)	Gain/Lost	Locus_tag#	Start	End	Gene
Xuzhou21	Lost	ECs2617	2593128	2594129	putative integrase
Xuzhou21	Lost	ECs2618	2594135	2594482	hypothetical protein
Xuzhou21	Lost	ECs2619	2594512	2595162	hypothetical protein
Xuzhou21	Lost	ECs2620	2595178	2595582	putative transcriptional regulator
Xuzhou21	Lost	ECs2621	2595672	2595809	hypothetical protein
Xuzhou21	Lost	ECs2622	2595881	2596084	putative DNA-binding protein
Xuzhou21	Lost	ECs2623	2596106	2596456	hypothetical protein
Xuzhou21	Lost	ECs2624	2596467	2596745	hypothetical protein
Xuzhou21	Lost	ECs2625	2596757	2596999	hypothetical protein
Xuzhou21	Lost	ECs2626	2597202	2597618	hypothetical protein
Xuzhou21	Lost	ECs2627	2597642	2597845	hypothetical protein
Xuzhou21	Lost	ECs2628	2597842	2598108	hypothetical protein
Xuzhou21	Lost	ECs2629	2598105	2598404	hypothetical protein
Xuzhou21	Lost	ECs2631	2598727	2598957	putative derepression protein
Xuzhou21	Lost	ECs2632	2599030	2599395	hypothetical protein
Xuzhou21	Lost	ECs2633	2599540	2602224	putative phage replication protein
Xuzhou21	Lost	ECs2634	2602301	2603260	putative plasmid partition protein
Xuzhou21	Lost	ECs2635	2603265	2603579	putative plasmid partition protein
Xuzhou21	Lost	ECs2636	2603599	2604288	putative transposase
Xuzhou21	Lost	ECs2637	2604288	2604614	putative transposase
Xuzhou21	Lost	ECs2638	2605245	2605721	hypothetical protein
Xuzhou21	Lost	ECs2639	2605974	2606462	putative tail protein
Xuzhou21	Lost	ECs2642	2609265	2609501	putative phage tail protein
Xuzhou21	Lost	ECs2643	2609429	2609794	putative tail protein
Xuzhou21	Lost	ECs2644	2609849	2610361	putative tail tube protein
Xuzhou21	Lost	ECs2645	2610361	2611545	putative tail sheath protein
Xuzhou21	Lost	ECs2646	2611703	2612026	putative tail protein
Xuzhou21	Lost	metW	3669897	3669973	
Xuzhou21	Lost	metZ	3669787	3669863	
Xuzhou21	Lost	valX	3249580	3249655	
Xuzhou21	Gain	CDCO157_1148	1266275	1266457	NinE
Xuzhou21	Gain	CDCO157_1143	1264534	1264803	hypothetical protein
Xuzhou21	Gain	CDCO157_1142K	1261627	1264533	putative replication protein P
Xuzhou21	Gain	CDCO157_1142J	1260740	1261516	predicted replication protein
Xuzhou21	Gain	CDCO157_0284	311472	312362	putative transposase
Xuzhou21	Gain	CDCO157_0283	311149	311475	putative transposase

# Locus tags starting with EC and CDC are Sakai and Xuzhou21 genomes respectively.

On the other hand, Sakai lost only six genes but gained 99 genes. Most of the genes gained are phage related, including phage Sp18. Sixty six genes were gained by the common ancestor of Xuzhou21 and Sakai (Table S4). All except two genes are in five blocks of two to 15 genes which were likely to have been gained by single events. None of these genes gained were known virulence-related genes.

The novel plasmid pO157_Sal in Xuzhou21 has been reported previously by our group [24], and this plasmid was only observed in ST96, ST23 and ST98 strains [24]. pO157_Sal contains no known virulence genes [24]. Interestingly, a similar plasmid (pEC4115) has also been found in EC4115 [22]. Our comparison showed that the two plasmids differ substantially (Table S5). Only 21 of the 52 pO157_Sal genes are homologous to genes in pEC4115 with amino acid level identity ranging from 28% to 51%. The majority of the homologous genes are type IV secretion system (T4SS) genes and genes associated with plasmid function. We also performed a phylogenetic analysis of the plasmids using TraE which showed that both plasmids belong to the same cluster (Figure S4) but are distantly related. It should be noted that several other O157:H7 strains carry a T4SS and were grouped together based on the TraE (Figure S4). The TraE sequences were extracted from partially sequenced genome sequences [22], and it is not known whether the T4SS in these strains was also plasmid borne.

The pO157 plasmid, which is present in all O157:H7 strains, shares high similarity among the four strains (Figure 2C). Xuzhou21 has the smallest number of changes with two SNPs in non-coding regions, one single base insertion and two single base deletions. It is interesting to note that all changes occurred singularly in one of the plasmids only. Thus there were no parsimony informative changes to infer relationships among the four plasmids.

### Xuzhou21 induced high levels of cytokine response and *stx2* expression

**Figure 3:**
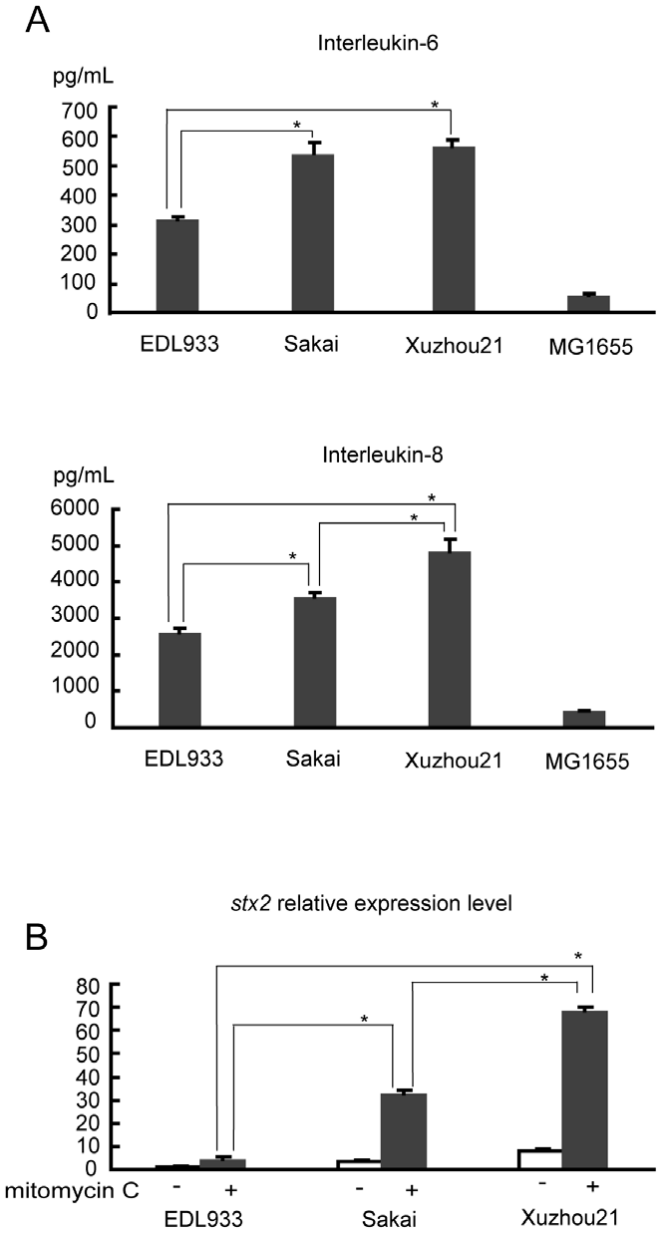
Comparison of the expressions of cytokines and *stx*2 among Xuzhou21, Sakai and EDL933. (A): The levels of cytokines (IL-6 and IL-8) of peripheral blood mononuclear cells response to 10^7^ heat-inactivated O157:H7 after four-hour incubation. All data were from three independent experiments. Differences were analyzed for significance by using the least significant difference (LSD) test following a significant *F*-test. Significant values relative to EDL933 (*P*<0.01) are indicated by a *. (B): Expression of *stx2*. The relative levels of expression under non-induction and induction conditions were relative to the expression level in EDL933 before induction by mitomycin C which was arbitrary set at 1.0. The relative value was averaged from three experiments. Error bars represent the standard errors. Differences in induction conditions were analyzed for significance by using the least significant difference (LSD) test following a significant *F*-test. Statistical significance for comparison of a given value with the value for EDL933 (non-induction condition) (*P*<0.01) is indicated by a *.

We determined whether there is a difference among the three strains Xuzhou21, EDL933 and Sakai in proinflammatory response as inflammation associated with HUS is marked by the release of cytokines/chemokines [27], [28], [29]. Using peripheral blood mononuclear cells (PBMCs), we assayed the level of production of two proinflammatory cytokines, IL-6 and IL-8. As shown in Figure 3A, the levels of expression of the two cytokines induced by Xuzhou21 and Sakai were significantly higher than that induced by EDL933 (ANOVA, *P*<0.01). Xuzhou21 also induced a significantly higher level of IL-8 than Sakai (*P*<0.01) while both induced similar levels of IL-6 (*P* = 0.36).

Previous studies suggest that the production of the Stx2 phage is associated with an increased risk of HUS [30]. We measured the level of the transcription of *stx2* in Xuzhou21, EDL933 and Sakai using real-time quantitative PCR. We first measured the *stx2* expression under non-inducing conditions. *stx2* was expressed constitutively in all three strains (Figure 3B) and its transcription levels in Sakai and Xuzhou21 were only 3.7 and 7.5 times higher than that in EDL933. As the Stx2 phage can be induced during infection leading to a much higher level of *stx2* expression [13], we used mitomycin C, a stimulus of the SOS response, for induction to determine levels of induced *stx2* expression *in vitro*. Relative to the constitutively expressed level in EDL933, induced *stx2* expression levels were 4.5, 32.7, 68.6 times higher in EDL933, Sakai and Xuzhou21 respectively and the difference was statistically significant (ANOVA, *P*<0.01). Interestingly we also observed more complete cell lysis in Xuzhou21 after three-hour Stx2 phage induction. Vero cell cytotoxicity assay was also performed. Xuzhou21 showed higher cytotoxicity after 12-hour incubation than EDL933 and Sakai (data not shown). We tested all other isolates in ST96 for induced expression of *stx2* and found that the isolates produced high levels of *stx2* with an average of 86.7 times higher than EDL933 and ranging from 36.6 times to 174.4 times, suggesting that elevated *stx2* expression is a general characteristic of the ST96 clone.

## Discussion

The outbreak of O157:H7 in Xuzhou, China, in 1999, led to 195 hospitalized HUS patients and 177 deaths, and is one of the largest O157:H7 outbreaks known, although it is hardly known outside China and was previously only reported in Chinese journals [6], [7]. From the epidemiological investigations, the outbreak was mainly associated with peasants living with animals carrying O157:H7 in the household, including goats, pigs, chickens and cattle. Courtyard animals carrying O157:H7 contaminated the surrounding environment through fecal shedding and persons who had poor personal and kitchen hygiene practice were more likely to be infected. It is well established that farm animals are carriers of O157:H7 [31], [32]. Additionally we found that 9% of the flies tested were positive for O157:H7 and thus they are important carriers in this outbreak. Flies may not just be mechanical vectors as O157:H7 can multiply inside the fly's mouth and be excreted through fly fecal matter [33]. Therefore poor hygiene and multiple routes of transmission may be the major contributing factors to the massive outbreak. However, increased transmission would have expected to increase number of infections but not higher number of HUS rate and high mortality rate. Host factors may contribute to higher mortality with a disproportional number of HUS cases and deaths in the older age groups (Table 2). We showed that the outbreak was caused by a new sequence type, ST96.

Genome sequence analysis of Xuzhou21 did not reveal any new virulence genes on its chromosome. However, Xuzhou21 carries an additional novel plasmid [24] which contains a T4SS and may contribute to the virulence of Xuzhou21. A recent study showed that the strain represented by TW14359 that caused the 2006 spinach-associated outbreak caused a much higher rate of HUS (15%) in comparison to other outbreaks in the US [2]. TW14359 has been shown to be on a divergent lineage from Sakai and EDL933. Comparative studies of TW14359 with other O157:H7 strains have revealed many genetic changes [13], [34], [35], but none of the strain-specific genes definitively contributes to HUS although a number of virulence factors were found [13].

Shiga toxin is a key virulence factor for O157:H7 pathogenesis [30], [36], [37] and indeed for any STEC infections including the recent O104:H4 outbreak in Germany [38], [39], [40]. The Stx1 and Stx2 prophages are present in all four genome sequenced strains and TW14359 also carries a Stx2c. Like Sakai and EDL933, the Stx1 and Stx2 prophages in Xuzhou21 were inserted in *yehV* and *wrbA* respectively. Kulasekara *et al.* [13] showed that TW14359 produced a 150 fold increase in Stx2 expression under mitomycin C induction and the induction was much higher than that occurred in EDL933. We also observed this differential inducibility between Xuzhou21 (and Sakai) and EDL933. The *stx2* expression in Xuzhou21 was 68.6 times of that under non-inducing conditions, twice of that induced in Sakai (32.7 times) and 15 times of that induced in EDL933 (4.5 times). Therefore, a crucial difference between the strains seems to be the differential inducibility of the *stx2* expression. A recent study compared the *stx2* expression of strains represent four different SNP clades using ciprofloxacin as the inducing agent and found that *stx2* was more highly induced in clade 8 strains including TW14359 than clades 1 to 3 strains [41]. Our finding that Xuzhou21 expresses a higher level of *stx2* than EDL933 is interesting as both Xuzhou21 and EDL933 belong to clade 3. There is clearly a large variation in *stx2* expression even within a clade. This observation also suggests that TW14359 and Xuzhou21 evolved to express higher levels of *stx2* independently.

The inducibility of *stx2* expression must lie within the Stx2 prophage genome as the Stx2 phage is inserted at *wrbA* in Xuzhou21 and Sakai, a different site (*argW*) from that in TW14359. We compared the four Stx2 prophage genomes (Figure S5) and found many differences among them, making it difficult to pinpoint the genetic determinants that exert the effect on *stx2* expression since both unique phage genes and nucleotide polymorphisms could affect *stx2* expression. Overall Xuzhou21 and Sakai shared more phage genes, consistent with whole genome relationship. The Q antiterminator upstream of *stx2* is crucial for *stx2* expression. However no variation was observed in this region among the four strains. Thus the observed phenotypic differences cannot be genetically linked to polymorphisms in the Q antiterminator genes, consistent with previous findings [42]. Antirepressors play an important role in phage induction [43]. One gene in the Stx2 prophage genome, ECs1199, encoding an antirepressor protein, was found to be absent in EDL933 but present in all three other genomes, in addition to an antirepressor (ECs1214) common to all four Stx2 prophages. We speculate this additional antirepressor regulates *stx2* induction and may contribute to the difference between Xuzhou21/Sakai and EDL933. We also examined other regulatory proteins including CI, CII, CIII, Cro, O and P. Variation in CI and Cro cannot be correlated with *stx2* expression differences observed since only Sakai varies in these two genes. CII, O and P are all polymorphic among the four strains with each strain being different whereas CIII is identical among all except EDL933 which differs by one amino acid. It is possible that variation in these genes differentially affect *stx2* expression.

The data of the two proinflammatory cytokines tested, IL-6 and IL-8, are consistent with the difference between EDL933 and Xuzhou21 (and Sakai). The levels of IL-6 and IL-8 of PBMCs induced by Xuzhou21 and Sakai were similar, but almost doubled the amount induced by EDL933. Although systemic inflammatory response does not seem to precede the development of HUS [44], there is a significant amount of data showing an association between inflammation and the development or the severity of HUS [27], [28], [29]. There is also an intricate direct relationship between cytokines and Shiga toxins. It is known that IL-6 and IL-8 are stimulated by Stx2 [45], [46], [47]. Xuzhou21 and Sakai induced much higher levels of *stx2* expression under mitomycin C induction, confirming this direct relationship of a highly inducible Stx2 phage with vigorous cytokine responses. Stx2 also induces production of other chemokines such as SDF-1α, SDF-1β, and RANTES [47] which simulate platelet function and renal thrombosis associated with HUS. Therefore these interactions further support the key role of *stx2* inducibility in virulence and disease development.

In conclusion, the 1999 China O157:H7 outbreak is so far the largest O157:H7 outbreak in terms of the number of HUS cases and deaths. We show in this study that the outbreak was caused by a new sequence type, ST96 and the outbreak strain Xuzhou21 is most closely related to Sakai. We found that Xuzhou21 has the capacity to provoke significantly elevated proinflammatory responses, and carries a highly inducible Stx2. Our analysis of Xuzhou21 provided further insight into the genetic basis of virulence of O157:H7.

## Supporting Information

Figure S1 **The temporal distribution of HUS cases of the 1999 outbreak in Jiangsu and neighboring Anhui province.**
(EPS)

Figure S2 **Phylogenetic tree of seven** ***E. coli*** **O157:H7 strains.** The phylogenetic tree is based on 902 intra- and intergenic SNPs identified as in figure 2B. Numbers on the branches were bootstrap values if >50% in percentage out of 1000 replicates. TW14588, EC4501 and EC4115 genome sequences were from Eppinger *et al.* [22].
(EPS)

Figure S3 **Alignment of the four genomes, Xuzhou21, Sakai, EDL933 and TW14359.** At top of each alignment is annotation of Xuzhou21 with locus_tag prefix CDCO157 and below four bands indicating how Xuzhou21, Sakai, EDL933 and TW14359 differ from the other genomes. Map positions in kb given below each genome. Large indels shown as blocks of colour (green shows deletion, red shows insertion) and gene names or locus tags are also shown for the indels not in Xuzhou21. SNPs are displayed as blue vertical lines. Single base insetions are in red and deletion in green. Regions which can't be aligned accurately were excluded and were indicated by a box.
(PDF)

Figure S4 **TraE tree.** The tree was constructed using Neighbor-Joining algorithm based on TraE amino acid sequences. The GenBank accession no. and protein sequences of TraE were described by Frank *et al.* [49] and Wang *et al.* [24]. The GenBank accession No. of other three *E. coli* O157:H7 strain are ACI39851 (EC4115 plasmid pEC4115), EDU72801 (EC4401) and ZP_03085718 (EC4024) respectively.
(EPS)

Figure S5 **Architecture of the Stx2 prophages.** The genomic location of the Stx2 prophage insertion and the locus_tag in the Xuzhou21 genome are indicated at the top row. Homologous genes are shaded in grey. The symbol *** is also used to indicate homologous genes when shading is not feasible. Corresponding genes are color coded: hypothetical genes in grey, transposase or integrase genes in blue, Shiga toxin 2 genes in pink, regulatory protein genes in red. For other genes, same color code was used if the genes have the same function. Regulatory protein CIII was present in EDL933 and TW14359 genome, but not originally annotated.
(EPS)

Table S1 **Recombinant segments in Xuzhou21.**
(DOC)

Table S2 **SNPs supporting the branching order of Xuzhou21 and Sakai.**
(DOC)

Table S3 **List of the fifty five SNPs unique to Xuzhou21.**
(DOC)

Table S4 **Genes gained by Xuzhou21 and Sakai.**
(DOC)

Table S5 **Comparison of pO157_Sal and pEC4115.**
(DOC)
