# Supplementary material for: A Novel Escherichia coli O157:H7 Clone Causing a Major Hemolytic Uremic Syndrome Outbreak in China
Source: PLoS One. 2012 Apr 27;7(4):e36144. doi: 10.1371/journal.pone.0036144 (PMC3338595; doi:10.1371/journal.pone.0036144)
Supplement: Table S1 — Recombinant segments in Xuzhou21. (DOC) [file pone.0036144.s006.doc]

**Table S1. Recombinant segments in Xuzhou21.**

| **No.** | **strain** | **Start** | **End** | **length** | **Gene** | **Gene_Start** | **Gene_End** | **Product** |
| --- | --- | --- | --- | --- | --- | --- | --- | --- |
| 1 | Xuzhou21 | 1260307 | 1268053 | 7747 | CDCO157_1142I | 1260278 | 1260577 | regulatory protein CII |
|  |  |  |  |  | CDCO157_1142J | 1260740 | 1261516 | predicted replication protein |
|  |  |  |  |  | CDCO157_1142K | 1261627 | 1264533 | putative replication protein P |
|  |  |  |  |  | CDCO157_1143 | 1264534 | 1264803 | hypothetical protein |
|  |  |  |  |  | CDCO157_1144 | 1264889 | 1265104 | hypothetical protein |
|  |  |  |  |  | CDCO157_1145 | 1265115 | 1265351 | hypothetical protein |
|  |  |  |  |  | CDCO157_1146 | 1265308 | 1265754 | hypothetical protein |
|  |  |  |  |  | CDCO157_1147 | 1265751 | 1266278 | putative DNA methylase |
|  |  |  |  |  | CDCO157_1148 | 1266275 | 1266457 | NinE |
|  |  |  |  |  | CDCO157_1148A | 1266732 | 1267409 | putative antirepressor protein |
|  |  |  |  |  | CDCO157_1149 | 1267484 | 1268206 | DNA-binding protein |
| 2 | Xuzhou21 | 1274211 | 1274398 | 188 | CDCO157_1158 | 1274231 | 1274476 | hypothetical protein |
| 3 | Xuzhou21 | 1799474 | 1799616 | 143 | CDCO157_1736 | 1796574 | 1800050 | putative host specificity protein |
| 4 | Xuzhou21 | 2120266 | 2120312 | 47 | intergenic |  |  |  |
| 5 | Xuzhou21 | 2177825 | 2177876 | 52 | CDCO157_2083 | 2179606 | 2177027 | putative portal protein |
| 6 | Xuzhou21 | 2758230 | 2758361 | 132 | CDCO157_2659 | 2758837 | 2760084 | multidrug efflux system subunit MdtA |
| 7 | Xuzhou21 | 3898252 | 3898335 | 84 | intergenic |  |  |  |
