# Supplementary material for: A Novel Escherichia coli O157:H7 Clone Causing a Major Hemolytic Uremic Syndrome Outbreak in China
Source: PLoS One. 2012 Apr 27;7(4):e36144. doi: 10.1371/journal.pone.0036144 (PMC3338595; doi:10.1371/journal.pone.0036144)
Supplement: Table S2 — SNPs supporting the branching order of Xuzhou21 and Sakai. (DOC) [file pone.0036144.s007.doc]

**Table S2. SNPs supporting the branching order of Xuzhou21 and Sakai.**

| **SNP_type** | **Strain** | **Xuzhou21** | **Site** | **Sakai** | **Site** | **EDL933** | **Site** | **TW14359** | **Site** | **Outgroup** | **Locus_tag** |
| --- | --- | --- | --- | --- | --- | --- | --- | --- | --- | --- | --- |
| **Xuzhou21/Sakai SNPs** | |  |  |  |  |  |  |  |  |  |  |
| intergenic | Xuzhou21/Sakai | t | 230035 | t | 230035 | c | 230036 | c | 230018 | c | CDCO157_23S01 |
| intergenic | Xuzhou21/Sakai | a | 346907 | a | 345586 | c | 345587 | c | 350778 | c |  |
| intergenic | Xuzhou21/Sakai | g | 3377287 | g | 3449652 | a | 3519495 | a | 3508638 | a |  |
| intergenic | Xuzhou21/Sakai | a | 4760323 | a | 4833571 | c | 4902335 | c | 4892746 | c |  |
| intergenic | Xuzhou21/Sakai | g | 4760324 | g | 4833572 | a | 4902336 | a | 4892747 | a |  |
| non synonymous | Xuzhou21/Sakai | t | 1268018 | t | 1264770 | g | 1350095 | g | 3209306 | g | CDCO157_1149 |
| non synonymous | Xuzhou21/Sakai | t | 1811319 | t | 1809380 | g | 1884324 | g | 1729946 | g | CDCO157_1745A |
| non synonymous | Xuzhou21/Sakai | c | 2640148 | c | 2710431 | t | 2786796 | t | 2617024 | t | CDCO157_2556 |
| non synonymous | Xuzhou21/Sakai | a | 3409620 | a | 3481985 | c | 3551524 | c | 3540970 | c | CDCO157_3257 |
| non synonymous | Xuzhou21/Sakai | t | 3798384 | t | 3871025 | g | 3938338 | g | 3930148 | g | CDCO157_3610 |
| non synonymous | Xuzhou21/Sakai | t | 4432275 | t | 4505017 | c | 4572246 | c | 4564163 | c | CDCO157_4207 |
| synonymous | Xuzhou21/Sakai | g | 1385906 | g | 1383967 | t | 1467739 | t | 1327839 | t | CDCO157_1254 |
| synonymous | Xuzhou21/Sakai | a | 1567499 | a | 1565556 | g | 1651678 | g | 1506757 | g | CDCO157_1478 |
| synonymous | Xuzhou21/Sakai | t | 1651066 | t | 1649126 | g | 1740911 | g | 1586085 | g | CDCO157_1581 |
| synonymous | Xuzhou21/Sakai | a | 1651087 | a | 1649147 | g | 1740932 | g | 1586106 | g | CDCO157_1581 |
| synonymous | Xuzhou21/Sakai | a | 1658968 | a | 1657028 | c | 1748813 | c | 1593987 | c | CDCO157_1590 |
| synonymous | Xuzhou21/Sakai | a | 1666258 | a | 1664318 | g | 1755724 | - | after_1599902 | g | CDCO157_1596 |
| **Xuzhou21/TW14359 SNPs** | |  |  |  |  |  |  |  |  |  |  |
| synonymous | Xuzhou21/TW14359 | t | 1268834 | a | 1265586 | a | 1350911 | t | 3210122 | t | CDCO157_1151 |
| non synonymous | Xuzhou21/TW14359 | a | 1268566 | g | 1265318 | g | 1350643 | a | 3209854 | g | CDCO157_1150 |
