# Supplementary material for: A Novel Escherichia coli O157:H7 Clone Causing a Major Hemolytic Uremic Syndrome Outbreak in China
Source: PLoS One. 2012 Apr 27;7(4):e36144. doi: 10.1371/journal.pone.0036144 (PMC3338595; doi:10.1371/journal.pone.0036144)
Supplement: Table S3 — List of the fifty five SNPs unique to Xuzhou21. (DOC) [file pone.0036144.s008.doc]

**Table S3. List of the fifty five SNPs unique to Xuzhou21.**

| **SNP_type** | **Xuzhou21** | **Site** | **Sakai** | **Site** | **EDL933** | **Site** | **TW14359** | **Site** | **Outgroup** | **Locus_tag** |
| --- | --- | --- | --- | --- | --- | --- | --- | --- | --- | --- |
| **Xuzhou21 SNPs** |  |  |  |  |  |  |  |  |  |  |
| intergenic | t | 967121 | c | 966111 | c | 967766 | c | 971067 | c |  |
| intergenic | t | 986123 | c | 985113 | c | 986771 | c | 990069 | c |  |
| intergenic | a | 1160515 | g | 1159474 | g | 1248697 | g | 1164422 | g |  |
| intergenic | t | 1650759 | c | 1648819 | c | 1740604 | c | 1585778 | c |  |
| intergenic | c | 1650761 | t | 1648821 | t | 1740606 | t | 1585780 | t |  |
| intergenic | g | 1664255 | a | 1662315 | a | 1754110 | a | 1599274 | a |  |
| intergenic | a | 2018333 | g | 2067506 | g | 2141154 | g | 1981082 | g |  |
| intergenic | c | 2620431 | t | 2690721 | - | after_2769820 | t | 2597315 | t |  |
| intergenic | g | 2620438 | a | 2690729 | - | after_2769820 | a | 2597323 | a |  |
| intergenic | c | 2620439 | g | 2690730 | - | after_2769820 | g | 2597324 | g |  |
| intergenic | t | 2620448 | c | 2690739 | - | after_2769820 | c | 2597333 | c |  |
| intergenic | t | 3343613 | c | 3415976 | c | 3485814 | c | 3474964 | c |  |
| intergenic | c | 3469718 | t | 3542053 | t | 3609343 | t | 3601014 | t |  |
| intergenic | g | 3553181 | a | 3625511 | a | 3692809 | a | 3684634 | a |  |
| intergenic | a | 4200678 | g | 4273418 | g | 4340640 | g | 4332539 | g |  |
| intergenic | g | 4466688 | t | 4539430 | t | 4608412 | t | 4598575 | t |  |
| intergenic | a | 4551324 | c | 4624342 | c | 4693325 | c | 4683487 | c |  |
| intergenic | t | 5285586 | c | 5397809 | c | 5427812 | c | 2080281 | g |  |
| non synonymous | a | 94859 | t | 94859 | t | 94860 | t | 94840 | t | CDCO157_0085 |
| non synonymous | t | 226700 | c | 226700 | c | 226701 | c | 226681 | c | CDCO157_0201 |
| non synonymous | g | 274813 | c | 274807 | c | 274808 | c | 277368 | c | CDCO157_0239 |
| non synonymous | t | 525447 | g | 524127 | g | 524129 | g | 529306 | g | CDCO157_0480 |
| non synonymous | t | 671116 | c | 670105 | c | 669800 | c | 674973 | c | CDCO157_0589 |
| non synonymous | g | 843205 | c | 842195 | c | 843849 | c | 847059 | c | CDCO157_0735 |
| non synonymous | t | 1205484 | g | 1204443 | - | after_1275970 | g | 1209622 |  | CDCO157_1087 |
| non synonymous | a | 1419423 | g | 1417486 | g | 1501258 | g | 1361358 | g | CDCO157_1300 |
| non synonymous | c | 1792810 | t | 1790871 | - | after_1867515 | - | after_1695341 | t | CDCO157_1733 |
| non synonymous | c | 1792906 | g | 1790967 | - | after_1867515 | - | after_1695341 | g | CDCO157_1733 |
| non synonymous | a | 1851233 | g | 1849294 | g | 1924241 | g | 1815279 | g | CDCO157_1779 |
| non synonymous | t | 2255119 | c | 2304291 | c | 2379579 | c | 2209567 | c | CDCO157_2166 |
| non synonymous | c | 2673380 | t | 2743664 | - | after_2819497 | t | 2650268 | t | CDCO157_2578 |
| non synonymous | c | 2977415 | a | 3049330 | a | 3120599 | a | 3045563 | a | CDCO157_2870 |
| non synonymous | g | 3365677 | t | 3438041 | t | 3507879 | t | 3497028 | t | CDCO157_3215 |
| non synonymous | c | 3371596 | g | 3443961 | g | 3513798 | g | 3502947 | g | CDCO157_3218 |
| non synonymous | c | 3515392 | t | 3587722 | t | 3655020 | t | 3646892 | t | CDCO157_3355 |
| non synonymous | a | 3685791 | g | 3758341 | g | 3825649 | g | 3817464 | g | CDCO157_3505 |
| non synonymous | t | 3883822 | c | 3956462 | c | 4023777 | c | 4015586 | c | CDCO157_3694 |
| non synonymous | t | 3951105 | c | 4023845 | c | 4091061 | c | 4082969 | c | CDCO157_3757 |
| non synonymous | a | 4336615 | g | 4409357 | g | 4476584 | g | 4468475 | g | CDCO157_4135 |
| non synonymous | a | 4248751 | g | 4321490 | g | 4388714 | g | 4380611 | g | CDCO157_4049 |
| non synonymous | c | 4613951 | t | 4686969 | t | 4755950 | t | 4746107 | t | CDCO157_4389 |
| non synonymous | t | 5271357 | g | 5383580 | g | 5413583 | g | 2080281 | g | CDCO157_4947 |
| non synonymous | a | 5322716 | t | 5434945 | t | 5464938 | t | 2080281 | g | CDCO157_4988 |
| non synonymous | t | 5354211 | c | 5466439 | c | 5496433 | c | 2080281 | g | CDCO157_5017 |
| synonymous | t | 182007 | c | 182007 | c | 182008 | c | 181988 | c | CDCO157_0160 |
| synonymous | t | 710525 | c | 709514 | c | 709296 | c | 714382 | c | CDCO157_0619 |
| synonymous | t | 881505 | c | 880495 | c | 882143 | c | 885449 | c | CDCO157_0773 |
| synonymous | t | 1339689 | c | 1337750 | c | 1421522 | c | 1280314 | c | CDCO157_1218 |
| synonymous | a | 1455548 | g | 1453611 | g | 1537383 | g | 1397484 | g | CDCO157_1339 |
| synonymous | t | 1691128 | c | 1689189 | c | 1780595 | - | after_1621101 | c | CDCO157_1622 |
| synonymous | t | 2026575 | c | 2075748 | c | 2149396 | c | 1989324 | c | CDCO157_1927 |
| synonymous | g | 2317365 | a | 2366537 | a | 2441733 | a | 2271813 | a | CDCO157_2228 |
| synonymous | t | 2522294 | c | 2571465 | c | 2646676 | c | 2476740 | c | CDCO157_2430 |
| synonymous | a | 2867632 | g | 2939547 | g | 3010814 | g | 2935781 | g | CDCO157_2769 |
| synonymous | g | 3060185 | t | 3132100 | t | 3202056 | t | 3128333 | t | CDCO157_2937 |
