# Supplementary material for: A Novel Escherichia coli O157:H7 Clone Causing a Major Hemolytic Uremic Syndrome Outbreak in China
Source: PLoS One. 2012 Apr 27;7(4):e36144. doi: 10.1371/journal.pone.0036144 (PMC3338595; doi:10.1371/journal.pone.0036144)
Supplement: Table S4 — Genes gained by Xuzhou21 and Sakai. (DOC) [file pone.0036144.s009.doc]

**Table S4. Genes gained by Xuzhou21 and Sakai.**

| **Strain** | **Gain/Lost** | **Locus_tag** | **Start** | **End** | **Gene** |
| --- | --- | --- | --- | --- | --- |
| Xuzhou21/Sakai | Gain | CDCO157_1144 | 1264889 | 1265104 | hypothetical protein |
| Xuzhou21/Sakai | Gain | CDCO157_1145 | 1265115 | 1265351 | hypothetical protein |
| Xuzhou21/Sakai | Gain | CDCO157_1457 | 1554779 | 1555153 | putative crossover junction endodeoxyribonuclease |
| Xuzhou21/Sakai | Gain | CDCO157_1458 | 1555150 | 1555971 | putative antitermination protein |
| Xuzhou21/Sakai | Gain | CDCO157_1459 | 1556884 | 1556384 | hypothetical protein |
| Xuzhou21/Sakai | Gain | CDCO157_1460 | 1556836 | 1557021 | hypothetical protein |
| Xuzhou21/Sakai | Gain | CDCO157_1485 | 1572610 | 1573326 | putative major tail subunit |
| Xuzhou21/Sakai | Gain | CDCO157_1712 | 1778640 | 1778855 | putative holin protein |
| Xuzhou21/Sakai | Gain | CDCO157_1713 | 1778860 | 1779204 | hypothetical protein |
| Xuzhou21/Sakai | Gain | CDCO157_1714 | 1779255 | 1779788 | putative endolysin |
| Xuzhou21/Sakai | Gain | CDCO157_1715 | 1780059 | 1780628 | antirepressor protein |
| Xuzhou21/Sakai | Gain | CDCO157_1716 | 1780782 | 1781249 | endopeptidase |
| Xuzhou21/Sakai | Gain | CDCO157_1717 | 1781839 | 1781612 | hypothetical protein |
| Xuzhou21/Sakai | Gain | CDCO157_1718 | 1781881 | 1782246 | putative Dnase |
| Xuzhou21/Sakai | Gain | CDCO157_1720 | 1782209 | 1782352 | hypothetical protein |
| Xuzhou21/Sakai | Gain | CDCO157_1721 | 1782536 | 1783099 | putative terminase small subunit |
| Xuzhou21/Sakai | Gain | CDCO157_1722 | 1783096 | 1784757 | putative terminase large subunit |
| Xuzhou21/Sakai | Gain | CDCO157_1723 | 1784821 | 1786758 | major head protein/prohead protease |
| Xuzhou21/Sakai | Gain | CDCO157_1724 | 1786970 | 1789549 | putative portal protein |
| Xuzhou21/Sakai | Gain | CDCO157_1726 | 1789552 | 1789878 | hypothetical protein |
| Xuzhou21/Sakai | Gain | CDCO157_1727 | 1789888 | 1790238 | putative head-tail adaptor |
| Xuzhou21/Sakai | Gain | CDCO157_1728 | 1790235 | 1790681 | hypothetical protein |
| Xuzhou21/Sakai | Gain | CDCO157_1729 | 1790678 | 1791022 | hypothetical protein |
| Xuzhou21/Sakai | Gain | CDCO157_1730 | 1790952 | 1791797 | putative major tail subunit |
| Xuzhou21/Sakai | Gain | CDCO157_1731 | 1791803 | 1792177 | putative tail assembly chaperone |
| Xuzhou21/Sakai | Gain | CDCO157_1732 | 1792201 | 1792482 | putative phage tail protein |
| Xuzhou21/Sakai | Gain | CDCO157_1733 | 1792535 | 1795615 | putative tail length tape measure protein |
| Xuzhou21/Sakai | Gain | CDCO157_1734 | 1795608 | 1795949 | minor tail protein |
| Xuzhou21/Sakai | Gain | CDCO157_1735 | 1795949 | 1796386 | minor tail protein |
| Xuzhou21/Sakai | Gain | CDCO157_2002 | 2118279 | 2117602 | putative tail assembly protein |
| Xuzhou21/Sakai | Gain | CDCO157_2005 | 2119629 | 2118931 | putative minor tail protein |
| Xuzhou21/Sakai | Gain | CDCO157_2006 | 2119958 | 2119629 | putative minor tail protein |
| Xuzhou21/Sakai | Gain | CDCO157_2007 | 2122567 | 2119955 | putative tail length tape measure protein precursor |
| Xuzhou21/Sakai | Gain | CDCO157_2008 | 2122961 | 2122548 | putative minor tail protein |
| Xuzhou21/Sakai | Gain | CDCO157_2009 | 2123410 | 2122988 | minor tail protein |
| Xuzhou21/Sakai | Gain | CDCO157_2010 | 2124176 | 2123424 | hypothetical protein |
| Xuzhou21/Sakai | Gain | CDCO157_2011 | 2124579 | 2124184 | putative minor tail protein |
| Xuzhou21/Sakai | Gain | CDCO157_2012 | 2125109 | 2124576 | putative minor tail protein |
| Xuzhou21/Sakai | Gain | CDCO157_2013 | 2125477 | 2125124 | putative tail attachment protein |
| Xuzhou21/Sakai | Gain | CDCO157_2014 | 2125887 | 2125489 | DNA packaging protein |
| Xuzhou21/Sakai | Gain | CDCO157_2015 | 2126954 | 2125929 | major capsid protein |
| Xuzhou21/Sakai | Gain | CDCO157_2016 | 2127342 | 2127010 | major capsid protein |
| Xuzhou21/Sakai | Gain | CDCO157_2017 | 2128671 | 2127352 | minor capsid protein |
| Xuzhou21/Sakai | Gain | CDCO157_2018 | 2130253 | 2128652 | putative portal protein |
| Xuzhou21/Sakai | Gain | CDCO157_2019 | 2130456 | 2130250 | head-to-tail joining protein |
| Xuzhou21/Sakai | Gain | CDCO157_2021 | 2132898 | 2132353 | putative terminase small subunit |
| Xuzhou21/Sakai | Gain | CDCO157_2022 | 2133285 | 2133509 | hypothetical protein |
| Xuzhou21/Sakai | Gain | CDCO157_2023 | 2133905 | 2133591 | putative transcriptional regulator |
| Xuzhou21/Sakai | Gain | CDCO157_2024 | 2134827 | 2134369 | putative endopeptidase |
| Xuzhou21/Sakai | Gain | CDCO157_2025 | 2135554 | 2134985 | putative antirepressor protein |
| Xuzhou21/Sakai | Gain | CDCO157_2026 | 2136358 | 2135825 | putative endolysin |
| Xuzhou21/Sakai | Gain | CDCO157_2027 | 2136753 | 2136409 | hypothetical protein |
| Xuzhou21/Sakai | Gain | CDCO157_2028 | 2136964 | 2136758 | putative holin protein |
| Xuzhou21/Sakai | Gain | CDCO157_2029 | 2136972 | 2137133 | hypothetical protein |
| Xuzhou21/Sakai | Gain | CDCO157_2030 | 2139263 | 2137455 | hypothetical protein |
| Xuzhou21/Sakai | Gain | CDCO157_2060 | 2155994 | 2155104 | putative transposase |
| Xuzhou21/Sakai | Gain | CDCO157_2061 | 2156317 | 2155991 | putative transposase |
| Xuzhou21/Sakai | Gain | CDCO157_2070 | 2163876 | 2162653 | putative tail fiber protein |
| Xuzhou21/Sakai | Gain | CDCO157_2070A | 2164540 | 2163941 | putative outer membrane protein |
| Xuzhou21/Sakai | Gain | CDCO157_2724 | 2840342 | 2840668 | putative transposase |
| Xuzhou21/Sakai | Gain | CDCO157_2725 | 2840665 | 2841555 | putative transposase |
| Xuzhou21/Sakai | Gain | CDCO157_2726 | 2842664 | 2841558 | putative protease/scaffold protein |
| Xuzhou21/Sakai | Gain | CDCO157_2727 | 2843964 | 2842462 | putative portal protein |
| Xuzhou21/Sakai | Gain | CDCO157_2728 | 2844200 | 2843964 | hypothetical protein |
| Xuzhou21/Sakai | Gain | CDCO157_2729 | 2846296 | 2844173 | putative terminase large subunit |
| Xuzhou21/Sakai | Gain | CDCO157_2730 | 2846769 | 2846293 | hypothetical protein |
