# Supplementary material for: A Novel Escherichia coli O157:H7 Clone Causing a Major Hemolytic Uremic Syndrome Outbreak in China
Source: PLoS One. 2012 Apr 27;7(4):e36144. doi: 10.1371/journal.pone.0036144 (PMC3338595; doi:10.1371/journal.pone.0036144)
Supplement: Table S5 — Comparison of pO157_Sal and pEC4115. (DOC) [file pone.0036144.s010.doc]

**Table S5. Comparison of pO157_Sal and pEC4115**

| **Locus_tag in pO157_Sal** | **Start** | **End** | **Gene name** | **Function** | **Locus_tag in pEC4115** | **Homology to pEC4115 *** |
| --- | --- | --- | --- | --- | --- | --- |
| pO157_Sal_01 | 1 | 1065 | *repA* | plasmid replication protein RepA | ECH74115_A0001 | 33/57 |
| pO157_Sal_02 | 1066 | 1569 |  | hypothetical protein |  | unique |
| pO157_Sal_03 | 1774 | 2190 |  | hypothetical protein |  | unique |
| pO157_Sal_04 | 2284 | 2961 | *traB* | Conjugal transfer protein TraB | ECH74115_A0009 | 34/49 |
| pO157_Sal_05 | 2976 | 3293 | *traC* | Putative conjugal transfer protein TraC | ECH74115_A0010 | 31/52 |
| pO157_Sal_06 | 3354 | 3752 | *traD* | Putative conjugal transfer protein TraD | ECH74115_A0011 | 28/47 |
| pO157_Sal_07 | 3697 | 6240 | *traE* | **Conjugal transfer protein TraE** | ECH74115_A0012 | 41/62 |
| pO157_Sal_08 | 6008 | 6904 | *traF* | Putative conjugal transfer protein TraF |  | unique |
| pO157_Sal_09 | 6963 | 7127 |  | hypothetical protein | inter-geneic | 37/41 |
| pO157_Sal_10 | 7129 | 7920 | *traG* | Conjugal transfer protein TraG | ECH74115_A0013 | 38/53 |
| pO157_Sal_11 | 7941 | 8729 | *traH* | Conjugal transfer protein TraH | ECH74115_A0014 | 42/58 |
| pO157_Sal_12 | 8722 | 9948 | *traI* | Conjugal transfer protein TraI | ECH74115_A0015 | 39/56 |
| pO157_Sal_13 | 10025 | 11086 | *traJ* | Conjugal transfer protein TraJ | ECH74115_A0016 | 51/67 |
| pO157_Sal_14 | 11108 | 13129 | *traK* | Conjugal transfer protein TraK | ECH74115_A0017 | 39/57 |
| pO157_Sal_15 | 13145 | 13510 | *traR* | conjugal transfer protein TraR | ECH74115_A0018 | 40/54 |
| pO157_Sal_16 | 13596 | 13982 |  | hypothetical protein |  | unique |
| pO157_Sal_17 | 13873 | 16197 | *topB* | DNA topoisomerase III | ECH74115_A0022 | 40/57 |
| pO157_Sal_18 | 16295 | 16717 | *stpA* | DNA binding protein, nucleoid-associated |  | unique |
| pO157_Sal_19 | 16730 | 17074 |  | hypothetical protein |  | unique |
| pO157_Sal_20 | 17071 | 17382 |  | hypothetical protein |  | unique |
| pO157_Sal_21 | 17597 | 18415 | *mpr* | zinc metalloproteinase Mpr protein | ECH74115_A0025 | a premature stop in pEC4115 |
| pO157_Sal_22 | 18399 | 18986 |  | hypothetical protein |  | unique |
| pO157_Sal_23 | 18997 | 19200 |  | hypothetical protein |  | unique |
| pO157_Sal_24 | 19443 | 19784 |  | hypothetical protein |  | unique |
| pO157_Sal_25 | 19806 | 20021 |  | hypothetical protein |  | unique |
| pO157_Sal_26 | 20021 | 20521 | *yeaA* | nuclease |  | unique |
| pO157_Sal_27 | 20527 | 20835 |  | hypothetical protein |  | unique |
| pO157_Sal_28 | 20961 | 21248 |  | hypothetical protein |  | unique |
| pO157_Sal_29 | 21290 | 21583 |  | hypothetical protein |  | unique |
| pO157_Sal_30 | 21638 | 22081 |  | hypothetical protein |  | unique |
| pO157_Sal_31 | 22078 | 22485 |  | hypothetical protein |  | unique |
| pO157_Sal_32 | 22486 | 22689 | *hha* | Haemolysin expression modulating  family protein | | unique |
| pO157_Sal_33 | 22708 | 23118 |  | hypothetical protein |  | unique |
| pO157_Sal_34 | 23161 | 23406 |  | hypothetical protein |  | unique |
| pO157_Sal_35 | 23842 | 24003 |  | hypothetical protein |  | unique |
| pO157_Sal_36 | 24245 | 24457 |  | hypothetical protein |  | unique |
| pO157_Sal_37 | 28374 | 24763 | *nikB* | relaxase | ECH74115_A0037 | 34/53 |
| pO157_Sal_38 | 27383 | 27072 | *nikC* | relaxosome accessory protein | inside ECH74115_A0037 | 28/41 |
| pO157_Sal_39 | 28807 | 28349 | *nikA* | Relaxosome component | ECH74115_A0038 | 29/48 |
| pO157_Sal_40 | 29094 | 29507 |  | hypothetical protein |  | unique |
| pO157_Sal_41 | 29510 | 30322 | *traL* | Conjugal transfer protein TraL | ECH74115_A0041 | 49/71 |
| pO157_Sal_42 | 30370 | 30927 |  | hypothetical protein |  | unique |
| pO157_Sal_43 | 30960 | 31313 |  | hypothetical protein |  | unique |
| pO157_Sal_44 | 31653 | 31348 |  | hypothetical protein |  | unique |
| pO157_Sal_45 | 32258 | 31656 |  | hypothetical protein |  | unique |
| pO157_Sal_46 | 33393 | 32398 |  | hypothetical protein |  | unique |
| pO157_Sal_47 | 33842 | 33516 |  | hypothetical protein |  | unique |
| pO157_Sal_48 | 34096 | 33941 |  | hypothetical protein |  | unique |
| pO157_Sal_49 | 34646 | 34323 |  | hypothetical protein | ECH74115_A0007 | 33/46 |
| pO157_Sal_50 | 35259 | 34678 |  | hypothetical protein |  | unique |
| pO157_Sal_51 | 36424 | 35372 | *traA* | Conjugal transfer protein TraA | ECH74115_A0006 | 29/51 |
| pO157_Sal_52 | 36692 | 36444 |  | hypothetical protein | ECH74115_A0048 | 37/59 |

* % amino acid identity /similarity. only coverage >60% is showed.
