# Supplementary material for: A Novel Escherichia coli O157:H7 Clone Causing a Major Hemolytic Uremic Syndrome Outbreak in China
Source: PLoS One. 2012 Apr 27;7(4):e36144. doi: 10.1371/journal.pone.0036144 (PMC3338595; doi:10.1371/journal.pone.0036144)
Supplement: Figure S3 — Alignment of the four genomes, Xuzhou21, Sakai, EDL933 and TW14359. At top of each alignment is annotation of Xuzhou21 with locus_tag prefix CDCO157 and below four bands indicating how Xuzhou21, Sakai, EDL933 and TW14359 differ from the other genomes. Map positions in kb given below each genome. Large indels shown as blocks of colour (green shows deletion, red shows insertion) and gene names or locus tags are also shown for the indels not in Xuzhou21. SNPs are displayed as blue vertical lines. Single base insetions are in red and deletion in green. Regions which can't be aligned accurately were excluded and were indicated by a box. (PDF) [file pone.0036144.s003.pdf]

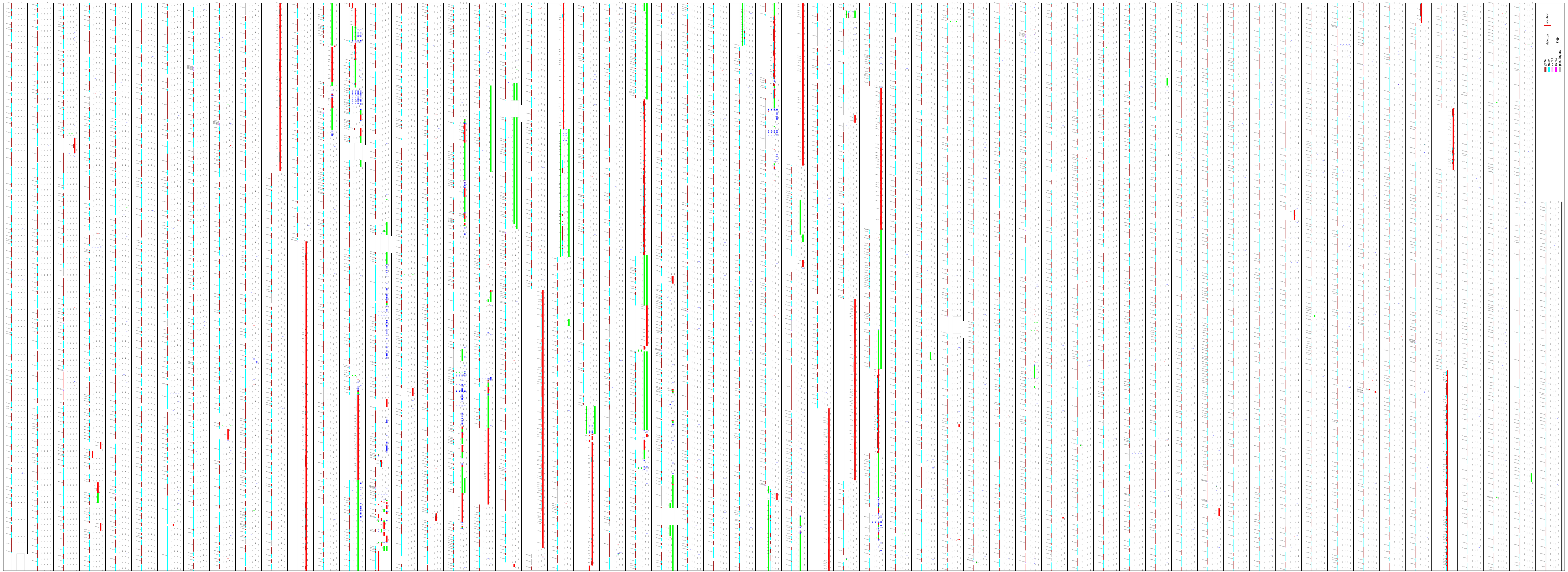

gene
gene
rRNA
tRNA
pseudogene
deletion
SNP
insertion
